# Developing BrapaCapture40K liquid chip for genetic research and breeding in *Brassica rapa*

**DOI:** 10.1093/hr/uhaf123

**Published:** 2025-05-14

**Authors:** Yifan Zhou, Liping Song, Lijie Zhong, Liguang Tang, Xueqing Zhou, Yuxian Zhu, Kun Wang, Aihua Wang

**Affiliations:** Wuhan Vegetable Research Institute, Wuhan Academy of Agricultural Sciences, College of Life Sciences, Wuhan University, Wuhan 430072, Hubei, China; State Key Laboratory of Hybrid Rice, College of Life Sciences, Wuhan University, Wuhan 430072, Hubei, China; Wuhan Vegetable Research Institute, Wuhan Academy of Agricultural Sciences, College of Life Sciences, Wuhan University, Wuhan 430072, Hubei, China; State Key Laboratory of Hybrid Rice, College of Life Sciences, Wuhan University, Wuhan 430072, Hubei, China; Wuhan Vegetable Research Institute, Wuhan Academy of Agricultural Sciences, College of Life Sciences, Wuhan University, Wuhan 430072, Hubei, China; Wuhan Vegetable Research Institute, Wuhan Academy of Agricultural Sciences, College of Life Sciences, Wuhan University, Wuhan 430072, Hubei, China; State Key Laboratory of Hybrid Rice, College of Life Sciences, Wuhan University, Wuhan 430072, Hubei, China; Institute for Advanced Studies, Wuhan University, Wuhan 430072, Hubei, China; Hubei Hongshan Laboratory, Wuhan 430070, Hubei, China; Wuhan Vegetable Research Institute, Wuhan Academy of Agricultural Sciences, College of Life Sciences, Wuhan University, Wuhan 430072, Hubei, China; State Key Laboratory of Hybrid Rice, College of Life Sciences, Wuhan University, Wuhan 430072, Hubei, China; Hubei Hongshan Laboratory, Wuhan 430070, Hubei, China; Wuhan Vegetable Research Institute, Wuhan Academy of Agricultural Sciences, College of Life Sciences, Wuhan University, Wuhan 430072, Hubei, China

Dear Editor,

*Brassica rapa* is among the most extensively cultivated vegetables worldwide, renowned for its immense agricultural and economic value. This species exhibits unparalleled intraspecific variation, spanning a spectrum of morphotypes such as Chinese cabbage, pak choi, turnip, mizuna, and choy sum [1]. These traits have also made it a popular horticultural crop, often cultivated in gardens and greenhouses. Consumer preferences for *B. rapa* breeding traits vary widely: compact Chinese cabbage varieties are in high demand, while turnips with smooth, tender, and crisp roots are favored, and choy sum is prized for its rapid growth and succulent stalks. To meet the ever-growing breeding demands for *B. rapa*, there is an urgent need for an economical and efficient high-throughput sequencing platform to accelerate both scientific researches and breeding innovations.

The development of DNA chips has become an indispensable strategy in modern breeding practices to cut the costs of sequencing and genotyping. While whole-genome sequencing remains cost-prohibitive for large-scale breeding populations, and conventional solid-phase chips exhibit limitations in both probe customization and sample throughput scalability—particularly demonstrating cost-inefficiency at lower sample volumes—liquid-phase capture technology offers distinct advantages by providing flexible probe design capabilities with significantly lower costs than sequencing platforms, while maintaining superior cost-effectiveness across various sample scales compared to array-based approaches. To date, SNP array chips or liquid-phase chips have been successfully applied to a wide range of crops such as apple, wheat, and soybean [2]. However, no DNA chip has yet been established for *B. rapa*. Here, we present BrapaCapture40K, a liquid chip-based genotyping tool that employs species-specific probes to efficiently capture target genomic sequences in *B. rapa*. This tool enables precise genotypic identification of target regions, paving the way for easy and fast genomic applications.

We downloaded whole-genome sequencing (WGS) data from a comprehensive collection of 384 *B. rapa* accessions encompassing nearly all recognized subspecies within the species (Table S1) [1, 3, 4]. Using the recently published AJ genome reference, we systematically selected 40 051 high-quality polymorphic loci through a three-phase process of structural filtering, linkage disequilibrium pruning, and adjacency refinement (Table S2) [4]. To ensure robust genotyping, centromeres, rDNA regions, and other high-repetition sequences were excluded during probe design (Fig. 1A). Of these loci, 1364 were specifically chosen to overlap genome-wide association study (GWAS) or QTL intervals derived from 27 studies (Table S3). The loci are distributed with a near-uniform pattern across gene-dense, non-repetitive regions, achieving an average interlocus spacing of 11.03 kb. Beyond unavoidable gaps in complex genomic regions, 30 249 loci (75.5%) are located within 10 kb of their neighboring loci, with a median distance of 5.08 kb. Remarkably, a significant portion (60.68%) of these loci is positioned within gene exons, introns, or promoter regions, collectively located in 20 609 (41.80%) and associated with 34 222 (69.40%) genes in the whole genome (Fig. 1B). These variants have median missing rates, heterozygosity and minor allele frequency (MAF) of 0.03, 0.08, and 0.31, respectively, reflecting their nature as predominantly nonrare variants with low missing and heterozygosity. Most pairwise linkage disequilibrium (LD) r^2^ values between adjacent variants are <0.2, supporting the notion that these loci are largely independently segregated (Fig. 1C). Moreover, the allele frequency distribution of these variants demonstrated a high degree of polymorphism, signifying their capacity to deliver substantial genetic insights (Fig. 1D).

**Figure 1:**
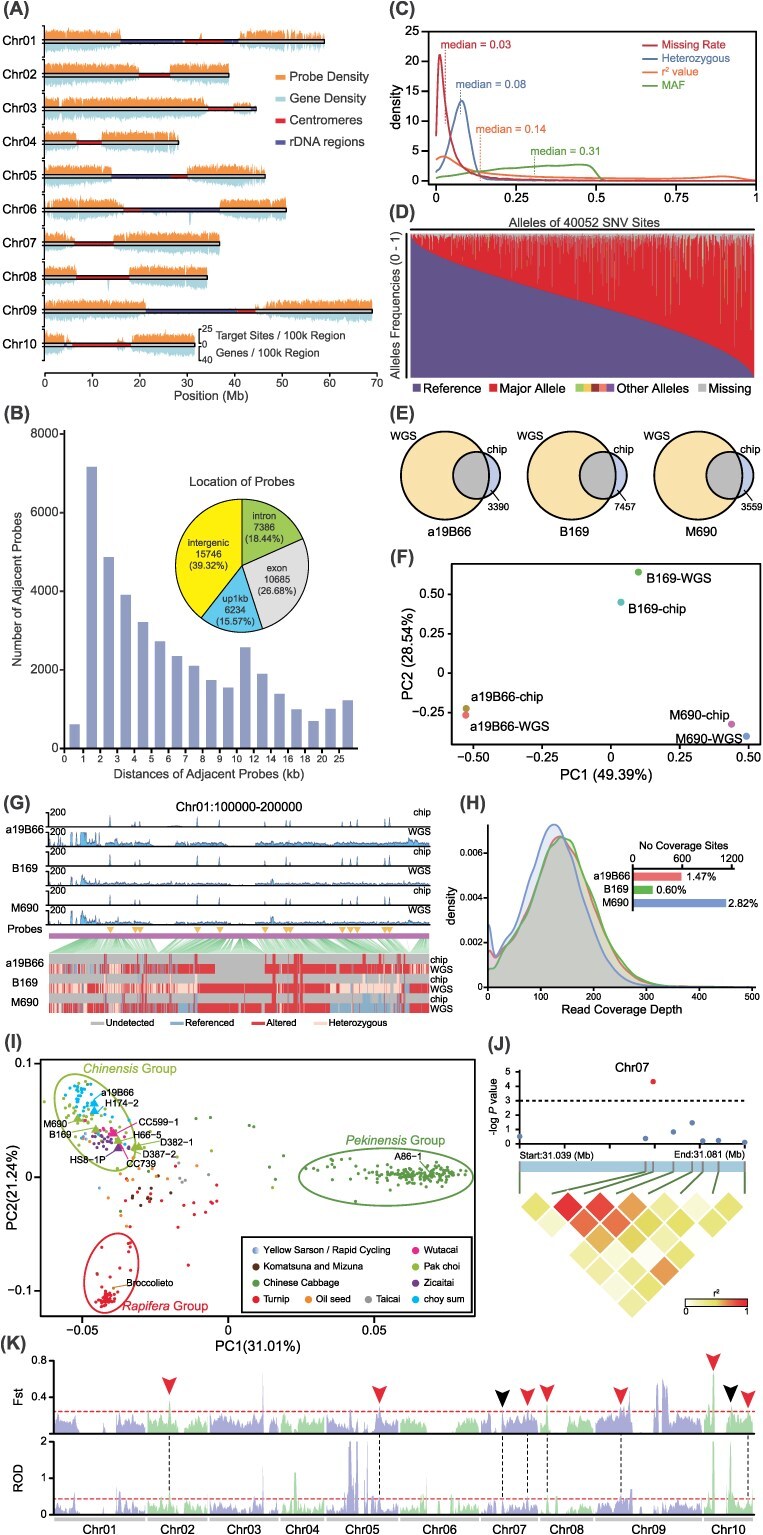
Character and application of BrapaCapture40K. (A) Density of genes and designed probes across the genome in 1-Mb windows. (B) Number of adjacent probe pairs across different distance categories, with the subpanel showing the location of probes relative to nearby genes. (C) Distribution of missing rate, heterozygosity, MAF of selected polymorphic loci, and LD *r^2^* value between adjacent loci. (D) Allele frequency for all selected polymorphic loci across 384 *B. rapa* accessions. (E) Venn diagram depicting the number of identified variants in the three samples, with ‘chip’ indicating variants genotyped by BrapaCapture40K. (F) PCA of the three samples sequenced by WGS and BrapaCapture40K, respectively. (G) Evaluation of sequencing and genotyping performance using BrapaCapture40K in a specific genomic region. The upper panel shows sequencing depth, while the bottom panel illustrates the identified polymorphic loci and their respective alleles. Probe locations are indicated by triangular markers, and the ‘chip’ represents data obtained from BrapaCapture40K. (H) Distribution of read coverage depths at probe sites across three chip samples, with the number of uncovered probe sites shown in the subpanel. (I) PCA of the 384 *B. rapa* accessions based solely on probe sites, with the positions of 11 additional test samples marked by triangles. (J) Zoomed-in GWAS results of *purpuraria* coloration trait associated with *BrMYB2*. (K) Reduction of diversity (ROD) and fixation index (Fst) values calculated with *purpuraria* accessions and non-*purpuraria* accessions as the two groups, using sliding windows with 1 Mb window size and 100 kb step size. Dashed lines indicate the top 5% threshold, and the arrows highlight regions overlapping with previously published results.

To assess the practical performance of BrapaCapture40K, we sequenced three *B. rapa* samples using both BrapaCapture40K and WGS methods. The variants identified by BrapaCapture40K were nearly all subsets of those detected by WGS, with very few unique variants identified exclusively by BrapaCapture40K (Fig. 1E). Principal component analysis (PCA) revealed that the clustering was sample-specific rather than strategy-specific, indicating that BrapaCapture40K provided an accurate and efficient representation of genetic information while significantly cutting down on sequencing and analytical costs (Fig. 1F). As a demonstration, we randomly selected a window and examined the sequencing results in detail. We found that BrapaCapture40K generated clear read peaks at each probe site, with the genotypes identified at these loci being in close agreement with those from WGS, confirming that the probes effectively capture the genome sequences in the target regions (Fig. 1G). Overall, the detection rates for the probes in these three samples were 98.53%, 99.40%, and 97.18%, with the average coverage depth at these loci surpassing 100× (Fig. 1H). Given that some probe sites still failed to capture the target regions, we further investigated these sites and found that approximately one-quarter of these sites were also undetectable in the WGS data. These sites were characterized by more variants nearby and lower average depth in the WGS data. Upon reviewing the 54 loci absent in all three datasets, we found that most of these sites were either fully or partially absent in the three accessions. These findings suggests that structural variations at the probe sites are the primary cause of the failure to detect these loci with BrapaCapture40K.

Then we evaluated the capacity of BrapaCapture40K for population structure analysis. By retaining the variants from 384 accessions across 40 051 target loci, we simulated and reconstructed a population map (Fig. 1I). The samples were clearly clustered into three groups—*chinensis*, *pekinensis*, and *rapifera*—along with several intermediate types, consistent with published reports [1]. The 11 additional samples sequenced using BrapaCapture40K also aligned perfectly with our expectations. In terms of our attempt to locate functional loci associated with the *purpuraria*’s purple color trait through GWAS, we pinpointed a signal at the *BrMYB2* gene on chromosome 7, which has been reported to be involved in the purple trait of *purpuraria* (Fig. 1J) [4]. Next, to further assess its performance in domestication studies, we investigated *purpuraria* and identified 10 domestication loci, seven of which overlapped with previously published results (Fig. 1K) [4]. All the above findings have underscored the ability of BrapaCapture40K to perform population structure analysis, domestication studies, and functional gene discovery.

In summary, we developed BrapaCapture40K, the first DNA liquid chip designed specifically for *B. rapa*. The chip is built upon 40 051 high-quality polymorphic loci from a diverse set of 384 *B. rapa* accessions, with DNA capture probes carefully designed to specifically target and enrich the genomic sequences of these loci. This considerably reduces the costs associated with subsequent sequencing and analysis. Upon evaluation, BrapaCapture40K has demonstrated excellent performance, boasted a high capture efficiency, and suggested to be well suited for both research and breeding purposes. The chip enables efficient identification of trait-associated markers, allowing breeders to accelerate selection for key agronomic traits. The market demand for *B. rapa* cultivars is expanding rapidly, with a growing emphasis on multifarious varieties. As breeders face increasing pressure to meet these demands, tools like BrapaCapture40K are critical in enabling convenient genomic analyses. Specifically, BrapaCapture40K facilitates marker-assisted selection by providing high-density genotyping data to track desirable alleles in segregating populations, thereby shortening breeding cycles for developing improved cultivars. By enabling rapid characterization of genetic diversity across breeding materials, the chip empowers precise parent selection for hybrid combinations and identification of heterotic groups, which are fundamental processes in modern *B. rapa* breeding programs. BrapaCapture40K is poised to become a popular tool in driving both basic research and applied breeding, accelerating the progress of *B. rapa* cultivation, and ensuring its continued economic relevance.

## Data Availability

All the raw sequencing data related are archived at Genome Sequence Archive of China National Center for Bioinformation (https://www.cncb.ac.cn/) under the project PRJCA034964. The detailed information (Supplemental Table) of BrapaCapture40K can be downloaded at the OMIX database (https://ngdc.cncb.ac.cn/omix/) under the accession OMIX008727.
